# Pharmacologic rationale for the NK1R antagonist, aprepitant as adjunctive therapy in HIV

**DOI:** 10.1186/s12967-016-0904-y

**Published:** 2016-05-26

**Authors:** Jeffrey S. Barrett, Sergei Spitsin, Ganesh Moorthy, Kyle Barrett, Kate Baker, Andrew Lackner, Florin Tulic, Angela Winters, Dwight L. Evans, Steven D. Douglas

**Affiliations:** Divisions of Clinical Pharmacology and Therapeutics, The Children’s Hospital of Philadelphia Research Institute, Philadelphia, PA 19104 USA; Translational Informatics, Sanofi Pharmaceuticals, Bridgewater, NJ USA; Division of Allergy and Immunology, The Children’s Hospital of Philadelphia Research Institute, Philadelphia, PA 19104 USA; Drexel University (BS Expected 2019), Philadelphia, PA 19104 USA; Tulane National Primate Research Center, Covington, LA 70433 USA; Department of Pediatrics, Perelman School of Medicine, University of Pennsylvania, Philadelphia, PA 19104 USA; Flow Cytometry Core Laboratory, The Children’s Hospital of Philadelphia Research Institute, Philadelphia, PA 19104 USA; Department of Psychiatry, Perelman School of Medicine, University of Pennsylvania, Philadelphia, PA 19104 USA

**Keywords:** HIV, SIV, HIV associated neurocognitive disorder, Substance P, Neurokinin 1 receptor, Aprepitant, Inflammation

## Abstract

**Background:**

Many HIV infected individuals with suppressed viral loads experience chronic immune activation frequently developing neurological impairment designated as HIV associated neurocognitive disorder (HAND). Adjunctive therapies may reduce HIV associated inflammation and therefore decrease the occurrence of HAND.

**Methods:**

We have conducted in vitro, animal and clinical studies of the neurokinin 1 receptor (NK1R) antagonist aprepitant in HIV/SIV infection.

**Results:**

Aprepitant inhibits HIV infection of human macrophages ex vivo with an ED_50_ ~ 5 µM. When administered at 125 mg once daily for 12 months to SIV-infected rhesus macaques, aprepitant reduced viral load by approximately tenfold and produced anti-anxiolytic effects. The anti-viral and anti-anxiolytic effects occur at approximately the third month of dosing; and the effects are sustained throughout the duration of drug administration. Protein binding experiments in culture media and animal and human plasma indicate that the free fraction of aprepitant is lower than previously reported supporting usage of higher doses in vivo. The analysis of blood samples from HIV positive individuals treated for 2 weeks with aprepitant at doses up to 375 mg demonstrated reduced levels of pro-inflammatory cytokines including G-CSF, IL-6, IL-8 and TNFα. Decreased pro-inflammatory cytokines may reduce HIV comorbidities associated with chronic inflammation.

**Conclusions:**

Our results provide evidence for a unique combination of antiretroviral, anti-inflammatory and behavioral modulation properties of aprepitant in vitro and in vivo. These results provide robust support for a clinical exposure target above that recommended for chemotherapy-induced nausea and vomiting. Doses up to 375 mg once daily in HIV-infected patients still elicit sub-therapeutic exposure of aprepitant though effective plasma concentrations can be achievable by proper dose modulation.

## Background

Advances in combined antiretroviral therapy (cART) [1] have made considerable improvements in morbidity and mortality for HIV infected individuals turning HIV into a chronic disease with a growing numbers of people living with infection. HIV-infected individuals however continue to have a shorter life expectancy and a higher risk of cardiovascular events, liver and kidney diseases, metabolic disorders, bone disease, and now have malignancies compared to the earlier days of the AIDS epidemic. HIV-infected individuals often experience increased immune activation, inflammation and coagulation what is now recognized as key features of pathogenesis of chronic HIV infection even with viral loads suppressed by cART [2, 3]. In addition, almost 50 % of HIV-infected individuals will develop (HAND) despite treatment with cART [1]. HIV causes nervous system disease at all stages of infection with adverse effects on quality of life, adherence to medications, employment and survival. HAND is often undiagnosed and untreated in both adolescents and adults. Issues that confound the presentation and treatment of HIV-induced nervous system disorders include the increasing prevalence of drug-resistant HIV strains, increasing age of HIV-infected patients, and hepatitis C virus co-infection. The clinical presentation, underlying the pathogenesis and treatment of this expanding group of neurocognitive and neurobehavioral disorders represent the clinical spectrum for which agents to treat HAND are needed.

Multiple adjuvant therapies with various mechanisms of action have been studied in HIV associated neurocognitive disorder (HAND), but none so far have shown a clear positive effect [4]. Current research targets substance P (SP) and its preferring receptor NK1R signaling pathway which mediates the interaction between the immune system and the nervous system [5, 6]. Aprepitant, an antiemetic substance P antagonist is approved for prevention of chemotherapy induced nausea and vomiting (CINV) has features which also make it an attractive candidate to treat residual inflammation in chronic HIV infection [5, 6]. Aprepitant crosses the blood–brain barrier [7], has immune-stimulatory and anti-inflammatory properties [5, 6, 8, 9] and some studies suggest that it improves general well-being and reduce depression [10] though this is not universally accepted. Extensive data support the premise that SP–NK1R signaling is important in the pathogenesis of HIV [6, 11–13]. HIV-infected men and women have elevated plasma levels of SP [14, 15]. The addition of SP in vitro enhances HIV replication in blood-isolated monocyte derived macrophages (MDM) and T-cells and NK1R antagonists inhibit this effect [12, 16–20].

The clinical datum supporting aprepitant’s clinical utility is defined by an acutely-treated CINV indication [21] and a poorly defined therapeutic window in patients with depression [22]; data in HIV-infected patients generated thus far have been based on pilot investigations of subtherapeutic doses at an insufficient duration of therapy to detect a meaningful clinical response [23, 24]. Our overall objectives for this investigation were to examine the exposure targets from in vitro, ex vivo and in vivo pharmacology studies with the goal of defining the dose–response relationship which support immune-stimulatory and anti-inflammatory benefit across experiment type, mechanism of action and organ system and species studied. Specifically, protein binding experiments conducted in culture media used for infectivity experiments and in the monkey (SIV animal model) and human plasma to confirm/revise the dose requirements based on accurate determination of the target free fraction. Ex vivo samples from donor subjects treated with aprepitant were used to determine pro-inflammatory cytokine response and establish a preliminary concentration-effect relationship. Samples from depressed and non-depressed HIV-negative patients were assessed for concentration-effect relationship of HIV infection of human macrophages treated with aprepitant. An in vivo pharmacology study in chronically treated SIV-infected rhesus macaques was conducted to assess anti-viral and behavioral responses. These results were collectively analyzed to determine their projection of clinically meaningful exposures in patients with HAND by conventional and model-based extrapolation techniques.

## Methods

### HIV infection and depression: patients utilized for ex vivo analysis

The effect of aprepitant on HIV infection was assessed ex vivo using monocyte-derived macrophages (MDM) from subjects recruited in conjunction with the clinical study “Depression Antidepressants and HIV infectivity” supported by R01-MH082670 [25]. A total of 150 depressed and non-depressed HIV negative participants completed structured diagnostic psychiatric assessments (SCID) for the presence of depression, Hamilton depression rating (17-item scale) and medical evaluation. Monocytes from 125 individuals of this cohort were available for the current investigation. Of this sample population, 70 (56 %) were female, 78 (62 %) were African American and 79 (63 %) were depressed. Ages ranged from 18 to 58 years (median 32 years). Subjects were excluded for medical comorbidities, current substance addiction, or use of psychotropic or immunomodulatory drugs in the 4 weeks prior to assessment. Blood samples were collected from the antecubital vein and monocytes were isolated as described below. The study was sponsored by the National Institutes of Mental Health and approved by the IRB of the University of Pennsylvania.

The effect of aprepitant treatment on cytokine production in vivo was studied using blood samples from patients enrolled in two separate randomized, placebo-controlled, double-blind phase IB trials evaluating the safety and antiviral activity of aprepitant. Details of these studies have been previously described [23, 24]. The study was conducted at AIDS Clinical Trials Unit and the Clinical and Translational Research Center (CTRC) of the Hospital of the University of Pennsylvania in Philadelphia, PA, USA. All patients signed a written informed consent. The study was sponsored by the National Institutes of Mental Health, approved by the IRB of the University of Pennsylvania, the US Food and Drug Administration (IND#75,558) and registered in Clinical Trials.gov (NCT00428519 and NCT01300988).

### Cell isolation and aprepitant effect on cytokine production PD-1 expression and HIV infection ex vivo

For HIV infectivity experiments PBMC were isolated from freshly collected blood samples using Ficoll-Paque (GE Healthcare, Uppsala, Sweden) gradient. Monocytes were purified by adhesion as described [26, 27] and cultured in 48-well plates at a density of 0.25 × 10^6^/0.5 ml/well in DMEM supplemented with 10 % FBS for 7 days before HIV-1 infection. Macrophages from each subject were exposed to four experimental conditions (none, 1, 5 and 10 µM of aprepitant). 7-day-cultured MDM were incubated with aprepitant for 2 h before infection with HIV-1 Bal (NIH AIDS Reagent Program), 5 ng/well based on p24 antigen assay, corresponding to a MOI of 0.02 determined using MAGI cells expressing CD4 and CCR5 (NIH AIDS Reagent Program). Cells were incubated with virus overnight, and then extensively washed to remove unbound virus. The culture medium and the aprepitant were replaced twice weekly. At day 7 post HIV infection, cellular RNA was extracted from the MDM for assessment of HIV gag mRNA expression using real time RT-PCR assays.

For cytokine and PD-1 assessment experiments freshly isolated peripheral blood mononuclear cell (PBMC) purchased from the Human Immunology Core Facility of The University of Pennsylvania School of Medicine (Philadelphia, PA) were cultured in RPMI medium supplemented with 10 % FBS overnight. Some cultures were treated with SP (10 µM) aprepitant (10 µM) or DMSO (0.001 %, vehicle for aprepitant). Cytokine concentrations were measured in supernatants using multiplex assay 24 h after SP/aprepitant treatment (see below). For PD-1 expression SP and aprepitant were added at day 0 and PD-1 expression was monitored daily for up to 8 days using flow cytometry.

### RNA extraction and real-time RT PCR assays

Total RNA was extracted from MDM cells using RNeasy kit (Qiagen), and the potential DNA contamination was eliminated by on-column DNase digestion as instructed by the manufacturer (Qiagen). Total RNA (1 µg) was reverse transcribed using AffinityScript QPCR cDNA Synthesis kit (STRATAGENE, Cedar Creek, TX) with random primer as instructed by the manufacture. 1 µl of the resulting cDNA was used as a template for real-time PCR amplification. Primers and probes for real-time PCR were synthesized by Integrated DNA Technologies, Inc. (Coralville, IA) as following: intracellular HIV RNA was detected using primers to the gag region as described [28] sense-5′-CATGTTTTCAGCATTATCAGAAGGA, antisense-5′-TGCTTGATGTCCCCCCACT, probe- 5′-FAM-CCACCCCACAAGATTTAAACACCATGCTAA-Q. Levels of glyceraldehyde 3-phosphate dehydrogenase (GAPDH) were used for normalization: sense-5′-GTGGTCTCCTCTGACTTCAACA, antisense-5′-TGCTGTAGCCAAATTCGTTG, probe 5′-5′-FAM-CTGGCATTGCCCTCAACGACC-Q. PCR was performed using iQ iCycler system (Bio-Rad Laboratories, Inc., Hercules, CA). Corresponding fragments of assayed genes cloned in pGEM-T vector (Promega) were used to generate standard curves.

### Multiplex cytokine assay

Concentrations of pro-inflammatory cytokines and chemokines in supernatants of human PBMC were measured using human Cytokine/Chemokine Magnetic Bead Panel—Premixed 30 Plex, HCYTMAG-60K-PX30 (Millipore, Billerica, MA, USA) as recommended by the manufacturers.

### Soluble CD163 (sCD163) assay

sCD163 was measured by ELISA (Trillium Diagnostics, Brewer, Maine, USA) according to manufacturer’s recommendations.

### Non-human primates

All experiments involving non-human primates were conducted at the Tulane National Primate Research Center. All experiments were approved by the Tulane Institutional Animal Care and Use Committee (Protocol 3267-B00). The Tulane National Primate Research Center (TNPRC) is an Association for Assessment and Accreditation of Laboratory Animal Care International accredited facility (AAALAC #000594). The NIH Office of Laboratory Animal Welfare assurance number for the TNPRC is A3071-01. All clinical procedures, including administration of anesthesia and analgesics, were carried out under the direction of a laboratory animal veterinarian. Animals were anesthetized with ketamine hydrochloride for blood collection procedures. All possible measures are taken to minimize discomfort of all the animals used in this study. Tulane University complies with NIH policy on animal welfare, the Animal Welfare Act, and all other applicable federal, state and local laws. All animals were from the Specific Pathogen Free breeding colony at the TNPRC. All animals were pair-housed in one room. Pairs were housing in interconnected caged depending on body weight of individual animals. The room was maintained on a 12:12 h light:dark cycle. The ambient temperature remained between 64 and 72 °F. Subjects were fed commercial biscuits twice daily and had access to fresh, clean water ad libitum.

A pilot study in two groups of three male Rhesus macaques (*Macaca mulatta*) was conducted to assess the exposure attained following once daily dosing of 80 and 125 mg aprepitant administered orally for 14 days (n = 3 per dose group). The 80 and 125 mg doses correlate with the human doses recommended for CINV patients (125 mg orally 1 h prior to chemotherapy treatment (day 1) and 80 mg orally once daily in the morning on days 2 and 3). On a weight basis these doses represent approximately 8 and 12.5 mg/kg (weight range, 7–12 kg for the rhesus) compared to 1.14 and 1.79 mg/kg human doses (assuming a 70 kg man). Higher weight-based doses were chosen based on the results of infectivity experiments supporting in vivo exposure targets an order of magnitude higher than observed with current CINV dosing regimens. Blood was collected for dense PK sampling on days 1, 7 and 14 and trough samples were collected prior to dosing on days 3, 5, 7, 11 and 13.

For infectivity studies, eight male rhesus macaques were used. Animals were infected intravenously with 50 ng p27 of SIV. Following infection four animals were treated with aprepitant once daily dosing of 125 mg aprepitant administered orally for a year and four infected animals were left untreated. Aprepitant (commercial capsule contents) was mixed and co-administered with various food preparations to ensure dosing compliance. Food preparations included peanut butter, marshmallow cream and honey, disguised in non-citrus fruit. While formal stability and delivery experiments were not conducted, pilot bioavailability studies confirmed the robustness of aprepitant exposures following food co-administration. The improved bioavailability with food-based dosing in conjunction with oral aprepitant is consistent with preclinical experiments which project simulated exposures based on the commercial nanosized aprepitant formulation. In this evaluation, in silico model predictions were similar to observed in vivo pharmacokinetic data in the fed state [29]; other studies have shown the benefit of complexation strategies to enhance aprepitant solubility/dissolution [30, 31].

Blood was collected for PK sampling on days 1, 7 and 14 and trough samples were collected prior to dosing on days 3, 5, 7, 11 and 13. Viral loads were assessed at 2 and 4 weeks after infection and monthly thereafter. One subject in the SIV-infected control group was excluded from the analysis due to its atypical response to SIV inoculation. Behavioral data were collected using video cameras installed on walls opposite the caging. Videotaping was scheduled to avoid disruptions in daily feedings, routine husbandry and research procedures. Data were collected pre-infection, 2 and 4 weeks post-infection and monthly thereafter. Videotaped recordings were analyzed using Observer XT^®^ (Noldus IT). Animals were sacrificed and necropsied after 1 year.

### Bioanalysis and pharmacokinetics

A validated, liquid chromatography-tandem mass spectrometry method was used for the quantification of aprepitant plasma concentration in HIV infected animals and tissue culture media. The method utilized a simple sample-preparation procedure of protein precipitation with methanol. Chromatographic separation was performed on a reversed phase C8 column (Hypersil Gold, 50 mm × 2.1 mm, 3 μm) using a mobile phase composed of acetonitrile and water in 0.5 % formic acid through gradient elution. Electro-spray ionization in positive mode was incorporated in the tandem mass spectrometric detection. The lower limit of quantitation of aprepitant in human plasma was 1 ng/mL. Details of the method validation have been previously published [32].

### Protein binding study

Protein binding experiments in human plasma and various experimental media were used to assess in vitro potency of aprepitant in 10 % FBS solution. These experiments were conducted to assess the linkage between in vitro and in vivo exposure targets in order to more accurately project clinical dose requirements for aprepitant. Protein binding was assessed via ultrafiltration and detection of aprepitant in various biofluids was conducted via LC–MS/MS.

Ultrafiltration units (CorningR Spin-X UF; MWCO 10 K) and filtrate collection tubes were obtained from Corning (Tewksbury, MA). Control human plasma was obtained by centrifugation of blood obtained from CHOP blood bank. Stock solutions of aprepitant in methanol were added to human plasma (1 %, v/v) to provide plasma samples at a nominal concentration of 10 μg/mL. This was further diluted with human plasma to provide 5, 2 and 0.5 μg/mL solutions. Similarly, aprepitant at above four concentration were also prepared in 10 % FBS in DMEM or 10 % FBS in water. Samples were incubated at 37 °C for 30 min to ensure equilibrium was established. Following incubation, an aliquot of sample was removed for determination of the drug concentration post incubation. Triplicate 500 μL aliquots of the samples were added to the sample reservoir of the UF unit with filtrate collection tubes. All ultrafiltration units were centrifuged at 13,000 rpm (15,000×*g*) for 15 min at 4 °C. Sample reservoirs containing the retentate were removed while 25 μL of the filtrate from collection tubes were transferred to the 96 well plate format. Internal standard solution (500 μL) was added to each sample analyzed by the validated LC–MS/MS method described above. While non-specific binding to filtrate collection tubes and ultrafiltration apparatus were not formally studied via control samples, similar binding experiments were part of the formal method validation and found to be negligible. Hence, low recovery of the test compound was not anticipated.

Calculation of % of protein binding was as follows:

$${\text{Percent}}\;{\text{bound}} = 1 - \left( {{\text{C}}_{\text{p,f}} / {\text{C}}_{ 0} } \right)\left( {1 + {\text{NSB}}} \right) \times 100$$

where Non Specific Binding (NSB) = 1 − (C_b,f_/C_b,uf_). C_b,f_ is the average concentration in the filtered PBS (ultrafiltrate). C_b,uf_ is the average concentration in the unfiltered PBS (retentate). C_p,f_ is the concentration in the filtered matrix. C_0_ is the dosing concentration of the compound.

### Statistical analysis

Data have been analyzed via basic descriptive statistics with results from 3 to 5 independent experiments expressed as the mean ± SD in most cases as indicated in the figure legends. Evaluation of the significance of differences between parameters of interest was performed using one-way ANOVA with post-test analysis by Bonferroni’s Multiple Comparison Test or Student’s t test when appropriate.

## Results

### Effect of aprepitant on HIV infection in human MDM

**Fig. 1:**
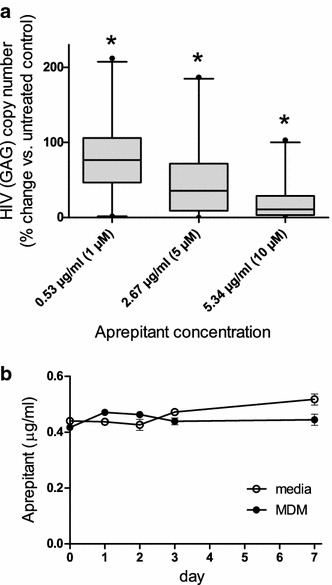
Aprepitant inhibits HIV replication in MDM. **a** Monocytes were isolated from the blood samples from 125 depressed and not depressed HIV negative subjects (for detailed description of donor population see “HIV infection and depression: patients utilized for ex vivo analysis”). Following differentiation for 7 days MDM were infected with HIV Bal. Macrophages from each subject were exposed to all four experimental conditions as indicated (1, 5 and 10 μM concentrations of aprepitant). Control, HIV infected MDM were treated with aprepitant vehicle, 0.001 % DMSO. RNA was collected 7 days after infection and assayed for expression of HIV GAG by real time RT PCR using the GAPDH housekeeping gene for normalization. GAG expression in control (non-treated) cultures were designated as 100 %; *p < 0.0001 between all groups including control were obtained through one-way ANOVA with post-test analysis by Bonferroni’s Multiple Comparison Test indicating significant difference between all groups in pair wise comparison. **b** Aprepitant added to DMEM 10 % FBS media or MDM cultures at a concentration of 1 μM (0.53 μg/ml) on day 0 and measured on days 0, 1, 2, 3 and 7. Results are presented as mean ± SD for three independent experiments

Aprepitant inhibited HIV infection in MDM from both depressed and not depressed HIV negative individuals ex vivo in a dose-dependent manner (Fig. 1a); *p* = 0.05 aprepitant 1 μM versus control and *p* < 0.001 aprepitant 5 and 10 μM versus control. A concentration of aprepitant of 10 μM is approximately equivalent to the IC_90_ value, and the IC_50_ value of aprepitant is about 5 μM. Depression status, sex, age or race had no effect on aprepitant inhibition of HIV infection in MDM (data not shown). Assay of aprepitant concentrations in culture media show little or no degradation during week long ex vivo, infectivity experiments (Fig. 1b).

### Aprepitant is highly protein bound

The protein binding study revealed that 99.9 % of aprepitant is protein bound. Human plasma and FBS ultrafiltration protein binding results from three experiments consistently show similar (99.79–99.95 %) binding of aprepitant to human and rhesus macaque plasma and FBS. These results suggest that aprepitant is more potent than previously appreciated; free drug concentration (responsible for aprepitant’s activity) is substantially less that the total concentration used to estimate IC_50_ in the in vitro experiments. Based on the free base aprepitant and converting to units of concentration based on aprepitant’s molecular weight yields a total IC_50_ concentration of 2.65 µg/mL. Correcting for protein binding yields a free drug IC_50_ concentration of ~2.65 ng/mL.

### Aprepitant exposure and effect on SIV infection in rhesus macaque

**Fig. 2:**
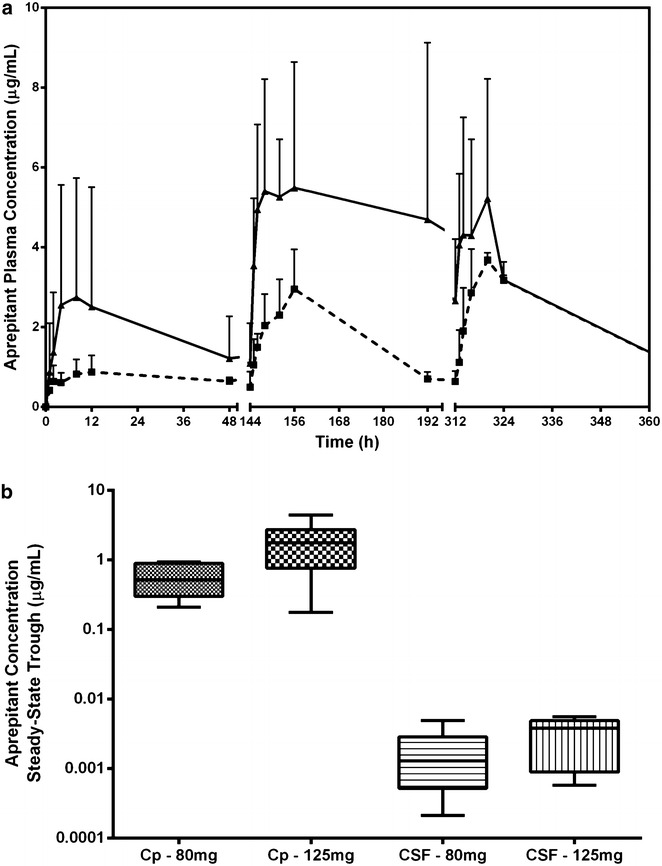
Aprepitant plasma and CSF concentrations in non-human primates. **a** Plasma aprepitant concentrations measured in rhesus monkeys (n = 3/group) following daily oral doses of 80 mg (*square*) or 125 mg (*triangle*). Data reflects mean aprepitant plasma concentrations on dense sampling days (1, 7 and 14) + SD. **b** Plasma (Cp) and CSF steady-state trough concentrations presented as *box-n-whisker plots*

Oral administration of aprepitant to non-human primates resulted in a dose dependent increase of aprepitant concentrations in plasma and CSF. Mean aprepitant plasma concentrations on the dense sampling days (1, 7 and 14) are shown in Fig. 2a. The distribution of steady-state trough concentrations for the 80 and 125 mg once daily dose groups are shown in Fig. 2b. The mean steady-state trough aprepitant concentrations in plasma were 0.6 and 1.9 µg/mL respectively, suggesting slightly greater than proportional increases in exposure over this dose range. The ratio of CSF to plasma concentration ranged from 0.05 to 0.8 % consistent with the high degree of protein binding and independent of dose. Trough plasma concentration is essentially reach steady state by day 10 in the rhesus while induction of CYP metabolism occurs by the second week of daily dosing. The extent of induction can be estimated by comparing the mean trough levels on days 13 and 14. The decline in trough exposure between these consecutive dosing days is approximately 32.5 % which is similar to that observed in the ferret and rat [33–35]. There were no differences in systemic and neuropathology between aprepitant treated and untreated Rhesus macaques.

**Fig. 3:**
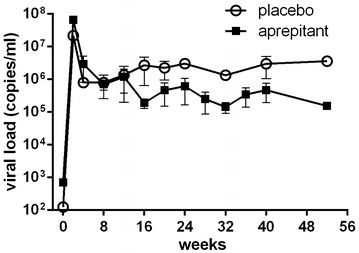
Plasma viral load in rhesus macaque* receiving 125 mg/day aprepitant * eight male rhesus macaques (*Macaca mulatta*) were infected with SIVmac251. Treated animals (n = 4/group) were administered 125 mg of aprepitant daily by the oral route, starting at day 0 for the duration of the study. Results are presented as mean ± SD for each group of animals

Based on the pilot study results yielding no side effects at a dose of 80 mg/day and sub-therapeutic exposure of aprepitant in plasma, we selected a dose of 125 mg/day for the infectivity study in rhesus macaque. Treatment of SIV infected rhesus macaques with aprepitant resulted in 1 log reduction in plasma levels of viral RNA as compared to non-treated controls (Fig. 3). Although reduction was not observed during acute infection (first 3 month), it was clearly evident during the 3–12 month observation period. No side serious effects were associated with 125 mg dose during the yearlong study.

**Fig. 4:**
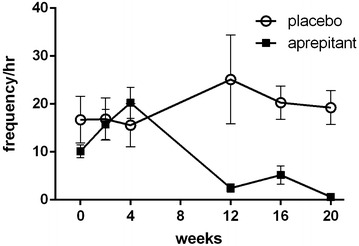
Anxiety-related behavior* time course in SIV infected rhesus macaque receiving aprepitant at 125 mg/day or placebo. *Anxiety-related behavior was calculated based on the following parameters: body shake, hypervigilant scanning, self-licking and self-grooming, scratching, unresponsive inactivity and yawning. Results are presented as mean ± SD for each group of animals (n = 4/group)

Monitoring of anxiety-related behavior for 20 weeks after infection demonstrated no significant difference between aprepitant treated and control groups during first 4 weeks after infection and significant reduction of anxiety thereafter (Fig. 4).

### Anti-inflammatory effect of aprepitant

**Fig. 5:**
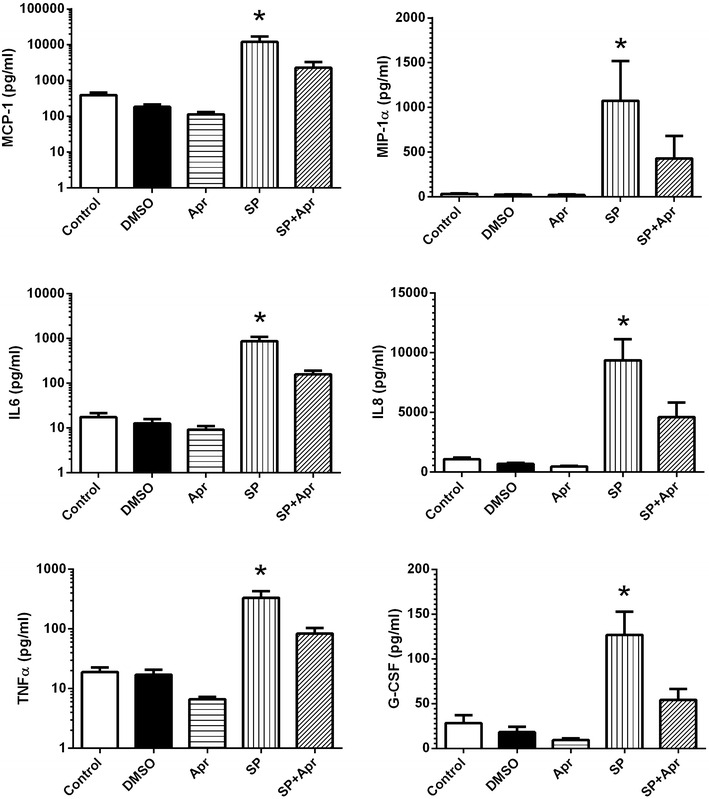
SP dependent up regulation of cytokine and chemokine production by human PBMC* is inhibited by aprepitant. *Freshly isolated PBMC from normal donors were incubated for 24 h with SP and/or Aprepitant (10 µM each) as indicated. Control cultures were either left untreated of incubated with DMSO (vehicle for aprepitant). Pro-inflammatory markers were measured in supernatants using MILLIPORE Human Cytokine/Chemokine Magnetic Bead Panel. Results are presented as mean ± SD for three independent experiments. *Statistically significant difference from control (*p* < 0.05)

**Fig. 6:**
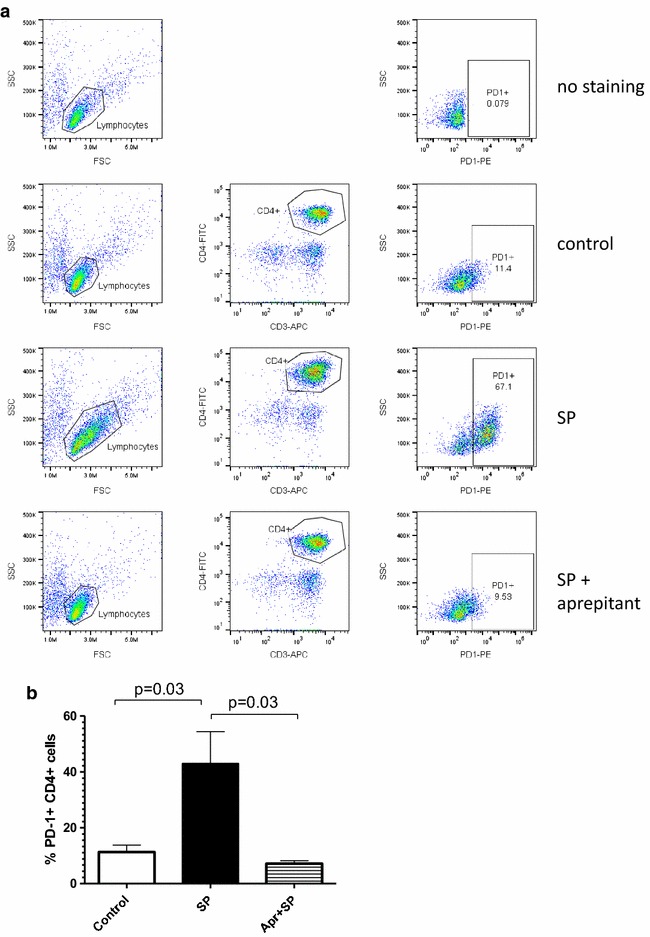
Aprepitant blocks SP induced PD-1 expression on CD4+ T-cells. PBMC were treated for 7 days with SP and aprepitant as indicated. PD-1 expression was measured by flow cytometry on CD3+CD4+ cells and expressed as percentage of PD-1 positive cells. **a** Results from representative experiment are shown. *First two columns* show gaiting strategy. *Last column* shows percent of PD-1 positive CD4 cells. **b** Mean results from five independent experiments and SD are shown; *p* values determined by paired Student’s t test. Median (spread): control 14.7 (11.1), SP treated 38.4 (60.6), SP + aprepitant treated 7.8 (6.0)

**Fig. 7:**
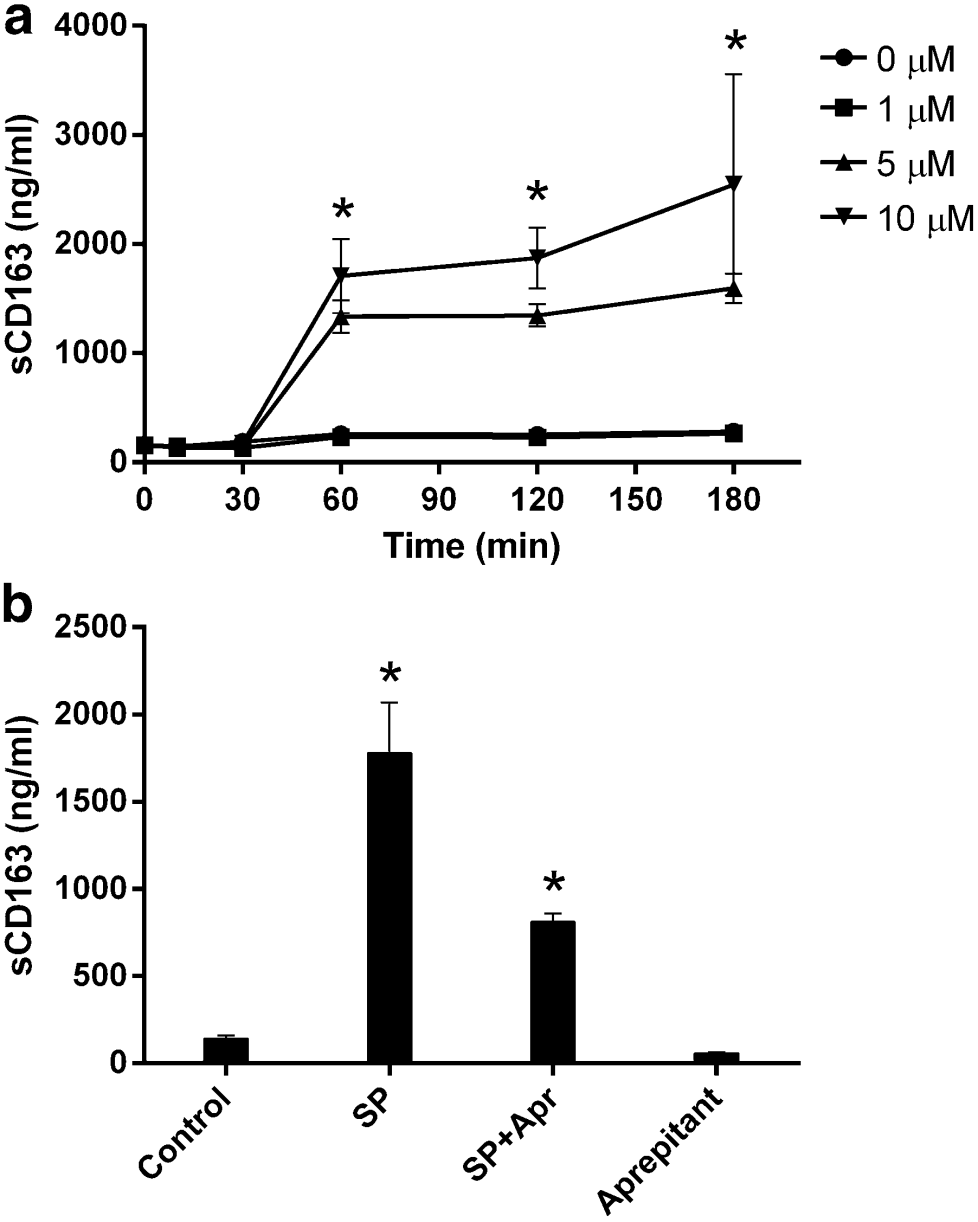
SP induces cleavage of CD163 in monocytes. sCD163 was measured by ELISA in the supernatant of freshly isolated human monocytes treated with SP and aprepitant as indicated. **a** Time course of cells treated with varying doses of SP as indicated. *p < 0.001 control versus 5 and 10 μM of SP. **b** Cells were pre-incubated with aprepitant (10 μM) for 30 min and then treated with SP (5 μM) for 60 min. *p < 0.001 SP versus control, *p* = 0.004 SP versus SP + aprepitant. Results are presented as mean ± SD from three independent experiments

Previously we reported that 2 weeks long daily administration of 375 mg of aprepitant to HIV positive individuals resulted in a decrease of serum levels of several pro-inflammatory cytokines, sCD163 and PD-1 expression on CD4 positive T-cells in vivo [24]. To evaluate mechanism and specificity of anti-inflammatory aprepitant action, PBMC from healthy donors were treated with SP in vitro with or without aprepitant present. Supernatants were analyzed using Multiplex inflammatory and immune response panel of cytokines and chemokines. SP upregulated production of the same cytokines and chemokines (G-CSF, IL-6, IL-8, TNFα and MIP-1α) which were down regulated by aprepitant in vivo (Fig. 5) [24]. In addition increased production of MCP-1 by SP was also detected. Aprepitant blocked the SP effect and DMSO had no effect. Incubation of PBMC with SP also resulted in increased PD-1 expression by CD4 T-cells (Fig. 6). The initial observation of increased percent of PD-1 positive CD4 T-cells was detected at day 4–5 (not shown) however a significant (*p* = 0.03) increase was observed after 6–8 days of exposure to SP (day seven results are shown in Fig. 6). The SP effect was blocked by aprepitant. Exposure of human macrophages to SP resulted in fast release of sCD163 in a time- and dose-dependent (*p* < 0.001) manner (Fig. 7) and this effect was also inhibited by aprepitant.

## Discussion

Our paper address two provocative questions: the viability of NK1R antagonism as a pathway for treating HIV-associated neurobehavioral and neurocognitive impairment and the suitability of aprepitant as a candidate molecule for this indication. We believe the data presented herein in addition to our and others’ previous findings support these contentions. This opinion, however, is counter to the common clinical belief which is heavily influenced by the use of aprepitant (Emend) for chemotherapy-induced nausea and vomiting (CINV) [36, 37] and failed clinical trials with aprepitant [38] and other NK1R antagonists in depression. However, this pathway may be clinically viable in depression at exposures eliciting complete saturation of the NK1R receptor (100 % occupancy) [39, 40] and the bridge to neurobehavioral benefits may be tied to the immunomodulatory and anti-inflammatory effects achieved [2, 5, 17, 41]. The salient point for this class and aprepitant specifically is that multiple therapeutic windows may exist to support target exposures for CINV and HAND.

Specifically, the effects of aprepitant on HIV infection in MDM extend our previous findings on the involvement of SP–NK1R signaling in HIV infection [8, 9, 19]. Using a large donor cohort we demonstrated inhibition of HIV infection in MDM ex vivo at a concentration of ~5 µM from 125 depressed and non-depressed HIV negative individuals which is line with projected free drug trough concentrations [42] required to elicit 100 % NK1R occupancy. Based on both ultrafiltration and equilibrium dialysis experiments more than 99.9 % of aprepitant was determined to be protein-bound when exposed to human plasma or 10 % FBS. These values are somewhat higher than the previously reported >95 % binding [43, 44] yielding free drug concentrations even lower than initially assumed. Likewise, the low CSF levels observed in monkeys confirm the low free fraction available to cross the blood brain barrier despite aprepitant’s lipophilicity. It should be noted that others have reported higher protein binding for aprepitant though not published formal results. Specifically, it has been observed and appreciated by regulatory authorities reviewing the original aprepitant submission [45]; these comments are likewise supportive of our results. The magnitude of the protein binding and the necessity of CNS penetration for both immunomodulatory and anti-inflammatory effects further supports the increased dosing requirements for aprepitant in HAND. Table 1 provides a broad overview of dosing requirements for similar agents across various therapeutic areas; in the majority of these agents daily dosing exceeded 600 mg/day. The only exceptions were agents with high bioavailability (>90 %) and where the systemic circulation was the site of action. Observed aprepitant exposure following chronic administration is reduced due to CYP 3A4-mediated enzyme induction across species and was confirmed in our primate study as well. Human trials in HIV-infected patients [23, 24] have yet to demonstrate the magnitude of the induction effect because of the short dosing durations over which drug dosing occurred (2 weeks) while previous studies suggest that induction occurs at approximately the third week of dosing following the once daily regimen (Merck & Co, reference briefing document, FOI [45]).

**Table 1:** Comparison of oral dosing requirements for representative highly protein bound drugs

Drug (PB %)	Class	Site of action	Effective conc	Dose
Clindamycin (95 %)	Antimicrobial	Respiratory tract, skin, soft tissue, and peritonitis	MIC susceptibility: medically significant pathogens *Staphylococcus aureus*: 0.016 μg/ml—>256 μg/ml *Streptococcus pneumoniae*: 0.002 μg/ml—>256 μg/ml *Streptococcus pyogenes*: <0.015 μg/ml—>64 μg/ml	Q6 h
Furosemide (91–99 %)	Diuretic	Kidney	Not well defined; varies based on disease state and desired effect	20–80 mg; repeat 6–8 h as needed; titrated up to 600 mg/d
Naproxen (99 %)	Anti-inflammatory	Peripheral tissues at site of inflammation	Trough plasma concentrations of 13–51 µg/mL at 500 mg Q12 h	250–1000 mg/d depending on indication and severity
Tolbutamide (96 %)	Hypoglycemic	Pancreas (beta cells)	ATP-dependent K+ currents of mouse pancreatic B-cells (patch-clamp technique): in the absence of albumin, tolbutamide blocked currents half maximally at 4.1 µmol/L	Initial dose: 1–2 g. Maintenance dose: 0.25–3 g daily
Efavirenz (99.5–99.75 %)	Antiretroviral	Throughout the body—site of infection and the reverse transcriptase enzyme	Plasma concentrations above 2 mg/L appear to suppress HIV replication	600 mg/d
Warfarin* (>98 %)	Anticoagulant	Blood	Effective concentrations influenced by many factors. Titrated based on INR response	Doses range from 0.5 mg daily to >20 mg; average dose 4–5 mg/d
Phenytoin (>90 %)	Anticonvulsant	Brain (motor cortex)	The clinically effective serum trough concentration is usually 40 to 80 µmol/L.	300 mg/d
Valproic Acid (90–95 %; saturable)	Anti-epileptic, Anticonvulsant	Brain	The therapeutic range for valproic acid (total) is 50–125 µg/mL	Doses vary by indication; average dose 500 mg/d; some patients benefit from doses up to 1000 mg/d
Diazepam+ (98.5 %)	Anti-anxiolytic	Brain/GABA_A_ receptor	Effective plasma concentrations 150–400 ng/mL	2–10 mg 2–4 times a day

* The site of action is the systemic circulation; only drug on the list with a bioavailability of 100 %

+ Bioavailability of 93 %; target concentrations <1 µg/mL

Yearlong treatment of SIV infected Rhesus macaques with aprepitant resulted in approximately tenfold reduction of viral loads observed between weeks 12 and 52 after infection. Coincidently, an anti-anxiolytic effect of aprepitant was observed during weeks 12 and 20 of therapy. It is clear from these results that a significant reduction in the behavioral effects observed in SIV-infected animals is elicited over time with daily administration of aprepitant. The magnitude of this effect is comparable to that observed in previous pharmacology studies in rhesus macaque with other anti-anxiolytic agents [46, 47].

In human studies with a 2-week 375 mg daily regimen in HIV positive individuals, we observed a reduction in several pro-inflammatory markers associated with poor HIV prognosis including PD-1 expression on CD4 T-cells, plasma levels of SP, and sCD163. We demonstrated a significant reduction in levels of G-CSF, MIP-1α, IL-6, IL-8 and TNFα after 2 weeks of aprepitant treatment [24]. These results are consistent with overall pro-inflammatory properties of SP [5, 6]. Reduction in plasma IL-6 levels is relevant since elevated IL-6 levels are linked to negative prognosis in chronic HIV [48]. In order to further evaluate these finding we measured SP and aprepitant effects in PBMC cultures from healthy HIV negative donors and found similar patterns of activation in vitro in comparison to human studies [24]. Treatment with SP increased production of the same cytokines and sCD163 with the exception of MCP-1 which changes were not observed in vivo, and increased frequency of PD-1 positive CD4 T-cells in vitro. SP effects in vitro were blocked by aprepitant. Chronic inflammation associated with HIV infection is also a likely cause of increased PD-1 expression and elevated plasma sCD163 levels in vivo. In this context it is not surprising that a compound with anti-inflammatory properties such as aprepitant reduces levels of those markers both in vivo and in vitro. Likewise, aprepitant’s composite effect also supports a role in the restoration of NK and T-cell functions, a potential therapeutic benefit in the treatment of chronic HIV.

While ex vivo experiments, non-human primate and human clinical studies indicate that NK1R antagonists may have limited use as monotherapy in HIV, they may be beneficial when used in combination with anti-retrovirals in chronic HIV given their anti-inflammatory effects and potential to improve neurocognitive functions through possible reduction in symptoms of depression and anxiety as well as improved sleep [40]. Defining the therapeutic dose of aprepitant in HIV requires a more definitive assessment of the target population and indication (HAND and/or related subgroups) as well as the potential comorbidities in these patients. In any case the dose is likely to be substantially higher than the 165 mg dose of aprepitant reported to achieve 97–99 % receptor occupancy in PET studies [7, 43]. The case for indication-specific dosing of NK1R antagonists has been previously discussed [39, 40], e.g., while partial receptor occupancy was sufficient to see improvement in insomnia, nearly 100 % occupancy may be required to see an effect in major depression [39], though a mechanistic rationale for these windows is still absent.

Other drugs and/or drug classes elicit multiple target exposure thresholds based on different clinical disease manifestations. This is the case for methotrexate, minocycline, thalidomide, prednisone and minoxidil and likely other well-known medicines. Likewise, these drugs have different dosing requirements for the various indications they seek to treat and, in many cases, there are well-designed clinical trials that support their registration in these indications as well as the specific dosing guidance. While we may have clearly defined mechanisms that support the separate disease targets (e.g., methotrexate for osteosarcoma vs. rheumatoid arthritis), in many cases exact pathways are less well understood. Interestingly, in the HIV arena, the drug efavirenz is only recently being appreciated for its multimodal actions despite its primary indication as part of the current HART standard of care. Specifically, efavirenz has been found to act as a 5-HT2A receptor partial agonist (Ki = 2.2 μM), 5-HT2C receptor ligand, serotonin and dopamine reuptake inhibitor (50 and 75 % inhibition at 10 μM, respectively), vesicular monoamine transporter 2 (VMAT2) inhibitor (60 % inhibition at 10 μM), and positive allosteric modulator of the GABAA receptor [49, 50]. It is generally believed that these properties, especially actions at 5-HT2 receptors, are involved in its neuropsychiatric adverse effects (depression, anxiety, hallucinations, aggression, suicidal ideation, and sleep disturbance), as they are alleviated by cyproheptadine, a drug with 5-HT2 receptor antagonist actions [49, 50].

Pro-inflammatory effects are a major contributor to co-morbidities in HIV. Our observed reduction in pro-inflammatory biomarkers (IL-6, IL-8, TNFα, sCD163, PD-1) provides evidence that potential synergy with cART may be attained via NK1R blockade. Any downstream effects with aprepitant would be expected to result in improved clinical consequences in both morbidity and mortality. Similar agents may be attractive for adjuvant therapy in neuro AIDS as well. Studies to evaluate the anti-inflammatory effects of aprepitant with longer treatment (4 weeks) and co-administration with ritonavir (CYP 3A4 inhibitor) to boost aprepitant plasma concentrations in virologically suppressed patients on cART are ongoing (Clinical Trials.gov # NCT02154360) but additional long-term trials will be essential to confirm proof-of-concept based on these promising supportive investigations.

## Conclusions

Our results provide evidence of a unique combination of antiretroviral, anti-inflammatory and behavioral modulation properties of aprepitant in vitro and in vivo. These results also provide robust support for a clinical exposure target above that recommended for chemotherapy-induced nausea and vomiting. Doses up to 375 mg once daily in HIV-infected patients still elicit sub-therapeutic exposure of aprepitant though effective plasma concentrations are likely achievable via proper dose. Proper modulation of the aprepitant dose will be an important factor in the overall success of this mechanism especially given the heterogeneity of the target population and the polypharmacy that defines the current standard of care.

Ethics approval and consent to participate

Human study was sponsored by the National Institutes of Mental Health, approved by the IRB of the University of Pennsylvania, the US Food and Drug Administration (IND#75,558) and registered in Clinical Trials.gov (NCT00428519 and NCT01300988). All patients signed a written informed consent. Non-human primate studies were approved by the Tulane Institutional Animal Care and Use Committee (Protocol 3267-B00). The Tulane National Primate Research Center (TNPRC) is an Association for Assessment and Accreditation of Laboratory Animal Care International accredited facility (AAALAC #000594). The NIH Office of Laboratory Animal Welfare assurance number for the TNPRC is A3071-01.

## Abbreviations

HAND: HIV associated neurocognitive disorder
NK1R: neurokinin 1 receptor
HIV: human immunodeficiency virus
SIV: simian immunodeficiency virus
G-CSF: granulocyte-colony stimulating factor
IL-6: interleukin 6
IL-8: interleukin 8
TNFα: tumor necrosis factor alpha
MIP-1α: macrophage inflammatory protein 1-alpha
cART: combined antiretroviral therapy
AIDS: acquired immune deficiency syndrome
CINV: chemotherapy induced nausea and vomiting
SP: substance P
SCID: structured diagnostic psychiatric assessments
PBMC: peripheral blood mononuclear cell
MDM: monocyte derived macrophages
PK: pharmacokinetics
FBS: fetal bovine serum
PD-1: programmed death
